# The Digital Platform and Its Emerging Role in Decentralized Clinical Trials

**DOI:** 10.2196/47882

**Published:** 2024-09-03

**Authors:** Rachel R Copland, Sten Hanke, Amy Rogers, Lampros Mpaltadoros, Ioulietta Lazarou, Alexandra Zeltsi, Spiros Nikolopoulos, Thomas M MacDonald, Isla S Mackenzie

**Affiliations:** 1 MEMO Research School of Medicine University of Dundee Dundee United Kingdom; 2 eHealth FH Joanneum Graz Austria; 3 Information Technologies Institute Centre for Research & Technology Hellas Thessaloniki Greece

**Keywords:** decentralized clinical trials, digital platform, digitalization, clinical trials, mobile phone

## Abstract

Decentralized clinical trials (DCTs) are becoming increasingly popular. Digital clinical trial platforms are software environments where users complete designated clinical trial tasks, providing investigators and trial participants with efficient tools to support trial activities and streamline trial processes. In particular, digital platforms with a modular architecture lend themselves to DCTs, where individual trial activities can correspond to specific platform modules. While design features can allow users to customize their platform experience, the real strengths of digital platforms for DCTs are enabling centralized data capture and remote monitoring of trial participants and in using digital technologies to streamline workflows and improve trial management. When selecting a platform for use in a DCT, sponsors and investigators must consider the specific trial requirements. All digital platforms are limited in their functionality and technical capabilities. Integrating additional functional modules into a central platform may solve these challenges, but few commercial platforms are open to integrating third-party components. The lack of common data standardization protocols for clinical trials will likely limit the development of one-size-fits-all digital platforms for DCTs. This viewpoint summarizes the current role of digital platforms in supporting decentralized trial activities, including a discussion of the potential benefits and challenges of digital platforms for investigators and participants. We will highlight the role of the digital platform in the development of DCTs and emphasize where existing technology is functionally limiting. Finally, we will discuss the concept of the ideal fully integrated and unified DCT and the obstacles developers must address before it can be realized.

## Introduction

In the last 2 decades, advances in digital health have given rise to clinical trial designs that are expected to disrupt the conventional clinical trial model [1-10]. Conventional clinical trials often depend on frequent in-person site visits, and are therefore limited to individuals who can easily access trial sites. The decentralized clinical trial (DCT), however, allows individuals to take part in research regardless of their geographical proximity to the trial site [11,12]. Moving trial activities away from sites and into participant homes or other local settings can also make trials less burdensome for participants [13].

DCTs are often, but not exclusively, facilitated by technology. Participants can be recruited through web-based advertising campaigns, receive study medications directly to their homes, self-report outcomes through smartphone apps, attend trial visits at home through videoconferencing software, and be monitored remotely with interconnected wearable devices [14-23]. Sponsors increasingly use these technologies as the pharmaceutical industry shifts toward using more decentralized or hybrid [24] methods [1,2]. To fully harness the benefits of this shift toward decentralization, we recommend the adoption of unified, integrated, and DCT-specific digital platforms.

A widely agreed definition of digital platforms does not yet exist; in this paper, we define them as software environments that facilitate the completion of clinical trial tasks. Digital platforms, hosted locally or on remote (cloud-based) servers, can also facilitate centralized data monitoring and oversight of trial activity, and typically adopt a modular architecture. A unified platform is one where disparate systems and modules centralize (or unify) to create a seamless user interface by linking systems to allow uninterrupted data flow. DCTs often incorporate several modes of data collection (eg, devices, electronic participant reported outcomes) from disparate sources. Unifying these data in a single platform avoids data sprawl while providing the opportunity for richer data insights and streamlined workflows [25,26]. The Eureka digital platform, for example, was developed by the University of California and has been used in several DCTs. The Eureka platform allows participants to consent, upload data, and attend remote trial visits. Meanwhile, trial staff can access real-time study progress data, generate reports, and collect analysis-ready data sets [27]. Eureka permits the customization of user interfaces with a granular role-based permissions system. Platforms like Eureka can be tailored to multiple users, including trial participants, researchers, sponsors, and third-party vendors, and are accessible on any suitable internet-connected device.

Although the use of digital platforms in DCTs is relatively common [27-34], existing platforms are not without limitations. Lack of interoperability and unclear data standardization protocols are known challenges to building unified systems [35-37]. Many proprietary digital platforms offer modules to support common DCT trial activities such as electronic data capture (EDC), drug dispensing, or remote visits. However, not all planned DCT activities may be available in a single proprietary system. To overcome this limitation, 1 option would be to integrate a third-party software solution that provides the necessary functionality often not permitted on proprietary systems. Even where third-party integration is allowed, subsequent software updates may be problematic [38]. Lack of data standardization leads to platforms and modules lacking interoperability, and custom implementations increase build complexity, extending timelines, and adding to costs.

With hybrid and decentralized study designs becoming more popular, the demand for innovative systems to support these trials is likely to grow; this will result in technology partners becoming important stakeholders in the success of DCTs, particularly for those studies requiring custom builds. The trend toward decentralization, however, has highlighted discordance between trialists and technology partners. Where technology partners specialize in full-stack development and user experience design, they are not experts on clinical trials. Likewise, trialists are adept at the intricacies of the clinical trial and DCT landscape, but they may harbor unrealistic expectations of system capabilities. This lack of shared understanding between stakeholders slows progress [39-41], and literature discussing platforms from both technical and clinical perspectives is scarce. We have attempted to bridge this gap in this viewpoint. We examine how digital platforms and DCTs have developed and highlight the advantages of a digital platform approach. We also discuss where user engagement in platform design can strengthen the DCT model. Finally, we demonstrate the key challenges of deploying a unified, modular, and fully-integrated platform in terms of technical limitations and data standardization.

## Digital Platforms: Then and Now

Early digital solutions for clinical trials took many forms, including EDC programs and software that helped streamline trial management. The INVEST trial compared treatment strategies for the treatment of hypertension in individuals with coronary artery disease [42,43]. The trial began in 1997 and was conducted using an internet-based software tool akin to a modern randomization and trial supply management system. Hosted on a Netscape Enterprise Server, the tool incorporated a database for real-time collection, verification, and validation of participant data at study visits. In the same year, Schoichet et al [44] described a communications network set up initially for the COMET trial, which investigated the efficacy of a chronic obstructive pulmonary disease treatment. The network included tools for data transfer, randomization and trial supply management activities, communications protocols, and an information access system for monitoring the trial status. The infrastructure was developed from preexisting local area networks, and frame relays were established between the participating organizations. An internal interactive voice response system was deployed to conduct and monitor tasks relating to drug supply management, collecting data in real-time. To monitor study activities, a platform was built on Lotus Notes (Lotus Development Corporation) with separate modules for enrollment of participants, trial monitoring, adverse event reporting, and tracking of case report forms.

Many modern platform interfaces, derived from early EDC systems, include participant-facing electronic diaries and web-based questionnaires. These have been used successfully in several early DCTs [45-47], and remain popular [32,48,49]. EDC platforms later expanded their capabilities beyond remote data capture to include other DCT elements, such as electronic consenting and telemedicine. Various DCTs have used the REDCap (Research Electronic Data Capture; Vanderbilt University) [50,51] electronic data capture platform to allow participants to upload trial data [52-54]. Other DCTs have used ResearchKit (Apple Inc.) [55], an open-source framework, to create participant platforms for users to consent, upload data, and view study results through a study-specific app [16,56,57].

There are several technology vendors offering ready-to-deploy, customizable, and often modular, platforms for DCTs. Companies such as IQVIA [58]; Medable [59]; Castor [60]; THREAD [61]; ObvioHealth [62]; Science37 [63]; Cognizant [64]; and AiCure [65], among others, offer digital platforms. For example, the RADIAL trial conducted by Trials@Home [66] is supported by eClinicalHealth’s Clinpal [67] digital platform. While conducting a recent systematic review of methods used to conduct DCTs, we found that, of 45 included trials, 31 managed their trials using a digital platform of some kind [24].

## Why Use Digital Platforms for DCTs?

The benefits of using digital platforms for DCTs extend to both participants and researchers. Digital platforms allow participants to be monitored remotely, which is associated with improved self-management of conditions and better clinical outcomes for participants [68-70]. Meanwhile, remote monitoring gives researchers greater insight into the day-to-day variability of disease activity and its wider impact [71,72]. Platforms with electronic participant reported outcome modules can increase participant satisfaction, improve data quality, reduce costs, and improve response rates [73-75]. There are also benefits associated with participants using their own devices to access study platforms. Directly uploading one’s information to the platform (ie, active data collection) offers participants greater flexibility and enables “on the go” or real-time reporting. On the other hand, data collected passively through devices, such as geolocation or screen interactions, provide researchers with greater insight into real-world behavior. The Brighten (Bridging Research Innovations for Greater Health in Technology, Emotion, and Neuroscience) DCTs, for example, actively and passively collected data to better understand the behaviors of participants with depression [76]. This combination of active and passive data collection could allow researchers to better monitor changes in behavior and, in theory, intervene sooner. DCTs with little to no participant-study team interaction may be experienced as disengaging by participants, but platform technologies can address these challenges. Push notifications, alerts, and reminders received through devices, phones, or personal computers have been cited as facilitators of engagement [77], and research has shown how these features have encouraged participants to complete study tasks and promote the use of study apps [78,79]. Research increasingly shows participants value having access to their own data [41,80], and displaying meaningful insights through participant dashboards could improve participant satisfaction. Another strategy for increasing engagement and promoting product longevity that is frequently adopted in commercial settings is gamification. Gamification incorporates game design elements in nongame contexts [81] to encourage reward-seeking behavior through incentives, such as unlocking new levels, receiving badges, or collecting points [82]. This strategy of promoting engagement has proved effective in several trials [83-88] and could be used to enhance both trial and platform engagement.

Digital platforms can be integrated with videoconferencing software, which allows participants to attend trial visits remotely [49,89,90]. Participant satisfaction with video visits is generally high, even when visits are completed on smartphones [91]. For instance, a platform could offer a web-based booking system, giving participants flexibility when scheduling follow-up visits. Further, integrating calendar application programming interfaces with the platform would allow visits to be added to the participant’s existing email or device calendars, which could be accompanied by pop-ups or reminders, potentially reducing missed or forgotten appointments. A combination of the tools mentioned above could generate a platform that promotes participant engagement with the trial while also allowing investigators to supervise trial progress and participant safety.

Conducting DCTs through digital platforms can also result in improved data integrity; this can be accomplished through platforms with data visualization dashboards, data tables, activity and audit logs, and real-time error flags, with unauthorized data edits restricted through access controls. Currently, most data visualizations and dashboards are implemented with web access in JavaScript or similar tools. More modern visualizations incorporate business intelligence (BI) approaches where different data sources can be combined and structured for quick visualization. BI tools can further help to find patterns and correlations between data. Data traceability can be achieved with rules-based sanity tests, semiautomated validation, and automated report generation [92]. Research increasingly supports the adoption of blockchain technology in clinical trials, with advocates suggesting that an immutable ledger using smart contracts would improve data integrity, accountability, and traceability [93-96]. For example, Liang et al [97], demonstrated a system whereby users controlled data access and protected privacy through blockchain technology and Intel software guard extensions. Data integrity and accountability were ensured by hashing protected personal health data and data access records and anchoring them to a long-lasting but secure ledger with platform dependencies. Building digital platforms on blockchain technology and other similar technological solutions warrants further exploration but could have significant implications for improving data integrity and security in DCTs.

## Anticipating Risks When Designing Platforms

### Overview

Maintaining digital security and compliance with local legislation and data standards are paramount to the success of DCTs, and digital platforms are not exempt from potential risks. While an unstable internet connection presents a source of weakness for data sharing, personal devices can also fall victim to malware or expose personal information if lost or stolen. As more trials embrace platform technologies, data security and governance risks will continue to grow [98]. Security and governance teams must, therefore, be established both at the Sponsor site and among all trial partners to scan for malware, apply patches to applications, and keep study personnel and participants informed on how to protect their data. As with conventional clinical trial approaches, DCTs are vulnerable to nonadherence and attrition. Learnings from mHealth studies show that users lose momentum with app use after a goal has been achieved and nothing new can be gained [99], which could have consequences for trials using digital platforms. Following best practice guidelines for DCTs, like choosing a research question of high perceived value [13] or offering flexibility in the DCT approach [100], may ameliorate some of these challenges, but research in this area is limited.

Finally, population demographics and individual preferences can impact how a user interacts with digital platforms, and anticipating these factors in advance can minimize later delays. For example, lack of digital literacy, inexperience with trial technology, insufficient digital infrastructure, and lack of confidence using devices are frequently cited reasons for withdrawal in app-based studies [101,102]. Embedding customization features into platforms may address this and encourage use, as has been demonstrated with mHealth applications [103-108]. Additionally, customization such as enlarged text functionality or text-to-speech embedded software would accommodate those with accessibility needs. Designing straightforward and simple user interfaces can avoid “overwhelm” and encourage regular use [99,109], and being able to access the platform on multiple devices allows participants to prioritize familiar devices. Age-related differences can influence how a user perceives the graphical user interface of the platform. Older adults perform better with classic, skeuomorphic interfaces (mimicking real-world objects), but younger adults may prefer the minimalism of flat design (clean and straightforward) [110]. Busy or cluttered platforms risk excluding individuals with dyslexia or visual impairments [111], potentially discouraging use [41,77], and improper use of color hues risks excluding color-blind individuals [112,113]. Further, research has shown that participants value simple interfaces [41,80] in digitally enabled DCTs. Additionally, users have preexisting expectations of platform functionality [114], (eg, “hamburger icons” denoting navigation menus), which should be considered in platform design. Typography, color, formatting, and layout can also influence user experience [115-118].

### The Challenge of Fully Integrated, Unified Digital Platforms

Many commercially available platform solutions are not yet open for integrating third-party components or are limited in their service provision and functionalities. Using existing components and already established solutions typically still results in a lack of interoperability, dependence on functionalities, and vendor lock-in. The future of digital platform technology, therefore, would look toward developing an end-to-end platform applying international standards where, if desired, users can integrate individual modules from different vendors.

Given the relative novelty of fully integrated, unified digital platforms for DCTs, specific interoperability standards for clinical trial platforms are not yet available. Nevertheless, commonly recommended standards are broadly applicable. We have summarized some of these standards in Table 1. The Healthcare Information and Management Systems Society [119] recommends interoperability standards in health care. These standards describe data sharing and offer recommendations regarding privacy and security. They define levels of interoperability, including technical and semantic. Future digital components used in DCTs should adapt and integrate these standards and recommendations for interoperability and modularity. As more data standardization processes become available, better ways to communicate and analyze data in trial platforms will become apparent [120].

**Figure 1:**
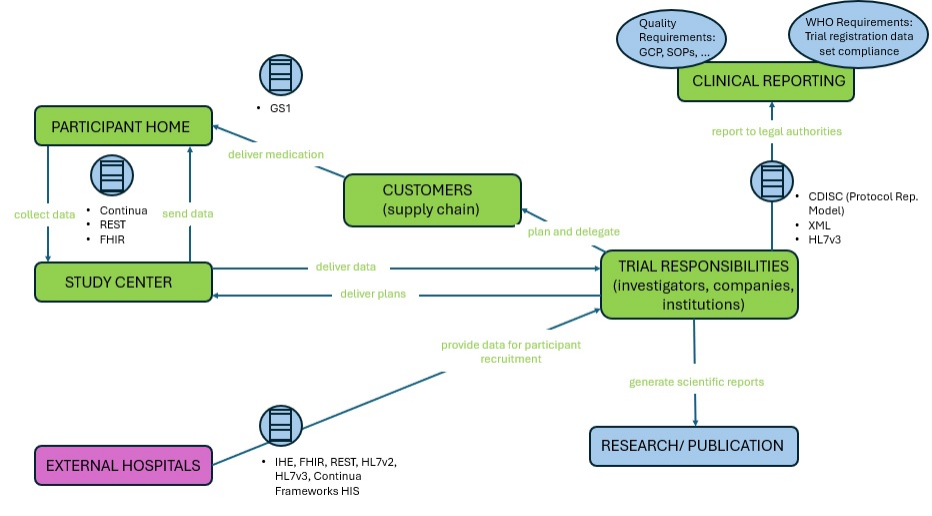
A reference architecture for components typically involved in decentralized clinical trials for data sharing and storage between participants’ homes and study centers. Data exists in a common format with preexisting standards to guarantee interoperability and modularity. Figure adapted with permission from Trials@Home Deliverable 2.4 [124]. CDISC: Clinical Data Interchange Standards Consortium; FHIR: Fast Healthcare Interoperability Resources; GCP: good clinical practice; GS1: Global Standards 1; HIS: hospital information system; HL7: Health Level 7; IHE: Integrating the Healthcare Enterprise; REST: representational state transfer; SOP: standard operating procedure; WHO: World Health Organization.

Figure 1 provides a reference architecture of components typically comprising a DCT. Architectures provide an abstract view of systems and can be used to manage communications and decisions. Domain, application, and site requirements lead to domain reference architectures which can be further refined to application architectures. These, in turn, define the final implementation architecture. The architecture comprising a digital platform is technically complex, and a further layer of complexity is added when integrating modules from external providers. Developing such a platform would first require defined system requirements (functional and nonfunctional), user requirements (user point of view, user goals, and user input and output), and business requirements (sponsor point of view, scope of trial, and business objectives). Within our reference model, data exists in a common format and the use of preexisting standards guarantees interoperability, but this exhibits a best-case scenario. Lack of interoperability represents a significant hurdle to the development of unified digital platforms, and the lack of specific data standardization processes may prevent innovation in this technology from being explored. Moreover, given the proprietary nature of many of the available commercial platforms, specific literature on integrations, architectures, data standardization, and general deployment strategies is restricted or unavailable. Despite these challenges, the development of a fully-integrated digital platform with in-built customization and tailoring is likely to provide suppliers with a competitive advantage in the growing DCT landscape.

**Table 1:** Existing data standardization processes and examples illustrating how these standards can be implemented in digital platform solutions.

Publisher	Data standard	Applicable use in digital platforms
HL7^a^	FHIR^b^	Profile released to store and transmit health data in a standardized, easy, and accessible format [121,122]. Digital platforms would benefit from using international standards for health data exchange partially already used in hospital information systems. HL2 version 3 promotes clinical document architecture which can be used to save clinical reports in a standardized form.
CDISC^c^	PRM^d^	Provides a standard for planning and designing a research protocol with focus on study characteristics such as study design, eligibility criteria, and requirements from ClinicalTrials.gov, WHO^e^, and EudraCT registries. PRM assists in automating case report form creation and EHR^f^ configuration to support clinical research and data sharing.
CDISC	CDASH^g^	Establishes a standardized way to collect data consistently across studies and sponsors so that data collection formats and structures provide clear traceability of submission data into SDTM^h^, delivering more transparency to regulators and others conducting data review.
CDISC	AdaM^i^	Defines dataset and metadata standards that support efficient generation, replication, and review of clinical trial statistical analyses, and traceability among analyses results, analyses data, and data represented in the SDTM [123]. Required standard for data submission to the FDA^j^ and Japan’s PMDA^k^.
GS1^l^	GS1 Global Data Model	Process automation in DCTs^m^. GS1 in health care is a global, voluntary group for all participants in the health care supply chain, which includes manufacturers, distributors, health care services, solution providers, regulators, and associations. A clinical trial electronic messaging standard implementation guide is provided [121].

^a^HL7: Health Level 7.

^b^FHIR: Fast Healthcare Interoperability Resources.

^c^CDISC: Clinical Data Interchange Standards Consortium.

^d^PRM: Protocol Representation Model.

^e^WHO: World Health Organization.

^f^EHR: electronic health record.

^g^CDASH: Clinical Data Acquisition Standards Harmonization.

^h^SDTM: Study Data Tabulation Model.

^i^ADaM: Analysis Data Model.

^j^FDA: United States Food and Drug Administration.

^k^PMDA: Pharmaceuticals and Medical Devices Agency.

^l^GS1: Global Standards 1.

^m^DCT: decentralized clinical trial.

## Conclusions

The growing popularity of DCTs necessitates an increasing reliance on technology. We believe the benefits of such technologies will be best realized through unified digital platforms. Platform approaches have historically been used to support DCTs, and the technology underpinning these platforms continues to develop. Tools for promoting trial engagement, maintaining investigator oversight, and improving data integrity can all be facilitated through digital platforms, and careful design planning helps accommodate the various needs of platform users. Digital platforms will likely evolve into singular, unified platforms that integrate external modules to generate a fully customizable system. However, challenges with poor interoperability and lack of data standards must first be addressed. Regardless of the current state of digital platforms for DCTs, the relationship between technology and trial innovation is likely to strengthen. Therefore, developing and implementing new DCT tools and solutions that support all stakeholders will require clinical and technical expertise.

## Abbreviations

DCT: decentralized clinical trial
EDC: electronic data capture
REDCap: Research Electronic Data Capture
